# The Dynamics of a Metapopulation: Changes in Life-History Traits in Resident Herring that Co-Occur with Oceanic Herring during Spawning

**DOI:** 10.1371/journal.pone.0102462

**Published:** 2014-07-22

**Authors:** Arne Johannessen, Georg Skaret, Lise Langård, Aril Slotte, Åse Husebø, Anders Fernö

**Affiliations:** 1 Department of Biology, University of Bergen, Bergen, Norway; 2 Institute of Marine Research, Bergen, Norway; Aristotle University of Thessaloniki, Greece

## Abstract

Different populations of Atlantic herring are regarded as forming a metapopulation, but we know little about the dynamics of the connectivity and degree of interbreeding between the populations. Based on data from three periods between 1962 and 2011, we identified the presence of two components of herring in a small semi-enclosed coastal marine ecosystem based on different somatic growth patterns and mean vertebrae sum (VS). The two components were interpreted as belonging to a resident herring population and the migratory, oceanic Norwegian spring spawning (NSS) herring population, and they co-occurred during spawning. In the 1960s, resident herring characterized by slow growth and low VS co-occurred with rapid growth, high VS oceanic NSS herring. Similar slow-growing resident and rapid-growing NSS herring were found in the 1970–80s, but both populations now had low VS suggesting similar origins. Finally, in the 2000s both populations showed rapid growth. The changes coincided with the NSS herring going from a state of high abundance and oceanic distribution to a collapse in the late 1960s that resulted in a coastal distribution closer to resident herring populations, before full recovery and resumption of the migratory, oceanic pattern in the 1990s. During all three periods, NSS herring were only present in the local system up to an age of about five years, but the synchronous spawning of the populations supports mixed spawning and interbreeding. During the investigation period both longevity, length at age (growth) and length-at-first maturity increased markedly for the resident herring, which then became more similar to the NSS herring. Genetic and/or cultural factors are believed to be the main causes of the observed changes in life history traits, although some effect of changes in environmental factors cannot be excluded. Our study suggests that relationships among populations in a metapopulation can be highly dynamic.

## Introduction

A metapopulation is defined as a set of populations with variable but moderate interbreeding [1], [2]. Atlantic herring is composed of several populations characterized by different life-history traits [3]–[5], but the gene flow between the different populations is believed to be sufficient to define them as belonging to the same metapopulation [6]. Several studies have shown that different herring populations occur together during the feeding season [7]–[9], but spatial overlap during the spawning period is obviously a prerequisite for interbreeding in a metapopulation.

In addition to the geographically widespread, migratory, oceanic Norwegian spring spawning (NSS) herring, a number of spatially segregated local populations of Atlantic herring exist along the Norwegian coast [10]–[14]. Local populations are highly dependent upon local processes, but are also influenced by external replenishment [15]. The semi-enclosed coastal marine ecosystem in Lindåspollene in south-western Norway harbours a resident herring population (LP herring) [12]. Herring of a different origin have been observed in the system [12], and Johannessen et al. [16] even reported that the same pre-spawning school contained both LP herring and another component with a similar maturation pattern.

To identify different components of herring with similar external appearance is challenging. Molecular methods are typically employed to obtain a “snapshot” of the contemporary genetic composition, but cannot for instance be used on lost historic material. The otolith appearance was utilised to separate different components in an earlier study in Lindåspollene [16], but a closer examination has shown that this analysis does not allow for correct classification of young herring. The growth pattern and the vertebrae sum (VS) have traditionally been used to separate different populations of herring [3], [5], [17]. VS are primarily determined by environmental factors during embryogenesis [18] and should therefore make it possible to distinguish the geographical origin of different components by statistical comparisons of average VS. The local populations, including that in Lindåspollene, have generally lower VS than NSS herring [14], [17], [19]. They have also evolved different life-history traits than the oceanic NSS herring and are generally characterized by slower growth, a shorter life span and higher relative fecundity [11], [12], [14], [17], [19]–[21].

There are several related factors that could influence connectivity among populations; the population size could influence the migration patterns that in turn could determine to what extent different populations interact. Changes in distribution range and use of spawning locations have been reported for several herring stocks in the North Atlantic following fisheries-induced population collapses [22]. The spawning stock of NSS herring dropped from more than 10 million tonnes in the 1940–50s to near extinction in the early 1970s [23] and rose again to full recovery in the late 1980s [24]. During this period the migration pattern changed from oceanic to coastal and back to oceanic [25]. In the 1950s, NSS herring had feeding and wintering grounds in the Norwegian Sea, and spawned off the western Norwegian coast. After the stock collapse, the remaining herring stayed along the coast throughout the year [26], [27]. After 1986, feeding migrations into the Norwegian Sea resumed [27], and spawning and overwintering areas gradually expanded northwards [26]. Hence, after the collapse, but prior to the recovery, the majority of NSS herring grew up along the coast and not in the traditional nursery areas in the Barents Sea [25]. Prior to the collapse, NSS larvae hatched from offshore banks and drifted into the fjords [25], [26], whereas the demersal spawning occurred further inshore after the collapse [25]–[28]. This distributional shift brought NSS herring closer to the resident populations along the coast [25], possibly increasing the likelihood of interbreeding. In addition, this opened up for transmission of culturally mediated migration patterns [29]–[32].

The earlier study of the population structure in Lindåspollene [16] was primarily based on data from only one year, and historical samples are crucial to an understanding of metapopulation dynamics [33]. There exists a long time series of data (1962–2011) on growth pattern and VS of herring in Lindåspollene in a period extending from pre-collapse to post-recovery of NSS herring with the potential to separate LP herring and any NSS herring occurring in Lindåspollene in a period of extremely variable abundance and distribution of the NSS herring.

We investigated the overlap in time and space between LP herring and immigrants from the migratory NSS herring as well as possible changes in life history traits in LP herring. We evaluated the potential for interbreeding by examining the co-occurrence of LP and NSS herring and the degree of synchronization of the maturation stages. We present the most plausible explanations for our observations, although other interpretations cannot be excluded.

## Materials and Methods

### Ethics statement

Most of the fish caught were dead when collected from the gillnets, with the few fish still alive being killed by a blow to the head and bending the neck to confirm death. Permission to catch wild fish and killing them by this method was given by the Directorate of Fisheries (Fiskeridirektoratet, Reguleringsseksjonen) and the County Governor of Hordaland.

### The Lindåspollene ecosystem

Lindåspollene is a small (∼7 km^2^) semi-enclosed ecosystem in south-western Norway comprising three 60–90 m deep basins (see [12]), with a main sill (7.5 m wide, 3.5 m deep) connecting to the outside fjord. The resident LP herring generally occur in two of the basins and play a key role in the system [12]. They are largely protected from harvesting [34].

The environment within the basins differs from that of the outside fjord (440 m deep with a sill at 20 m), in particular with regard to oxygen levels [12], [35]. There is low zooplankton biomass in the basins and a dominance of small copepods compared to the outside fjord [36], [37], [38]–[40], which may be due to generally poor oxygen conditions and limited inflow due to the shallow sill [41]. There are large annual variations in temperature but no long-term trends over the course of the study period [12], [35], [42]. No data are available for an adequate comparison of other environmental conditions in the course of the study period including abundance of potential herring prey. In the outside fjord, unlike in other studied Norwegian fjords, mesopelagic fish are lacking, and crown jellyfish, *Periphylla periphylla*, occur in high concentrations [43], [44]. As jellyfish are inefficient consumers of large zooplankton [44], this may have resulted in good feeding conditions for herring that migrated out of Lindåspollene. The development of the abundance of jellyfish and mesopelagic fish over our study period is not well documented. Fishermen observed jellyfish in varying amounts as early as in the late 1940s, and the first scientific study in the outside fjord reported high concentration of jellyfish in the early 1970s [45], [43].

### Biological sampling

Gillnet samples of herring were obtained from 1962 to 2011 in the three periods 1962–1964 (P1), 1970–1982 (P2) and 2005–2011 (P3) (see Table 1), where P1 coincides with the period prior to the eventual collapse of the NSS herring (year classes (YCs) represented: 1950–60s), P2 with the period during the collapse (YCs represented: 1960–1970s) and P3 with the period after the recovery (YCs represented: 1990s–2000s). Length and weight were recorded for nearly all fish and age for the majority of the fish. Meristic analyses (vertebrae sum, VS) were conducted throughout the period but with a low effort in the 1970s.

**Table 1:** Summary information of herring samples from Lindåspollene 1962–2011, with sampling month, total number of fish analysed (Total n), number of gillnet settings (n settings; referring to single gillnets from 2008 onwards and series of attached gillnets prior to 2008), number of fish sampled for age determination (Age) and VS.

Year	Month	Total n	n settings	Age	VS
1962	11	350	3	304	346
1963	3, 9–12	425	8	402	410
1964	1–7	809	14	747	478
1970	10–11	129	3	125	128
1971	1–11	978	13	925	566
1972	1,3–5, 8, 10–11	569	8	526	146
1973	1–3, 7, 9–10	266	8	258	50
1974	1–4	405	7	396	−
1975	3, 4	150	2	147	−
1977	3, 4, 9	751	9	652	−
1978	3, 4	340	7	331	−
1979	4, 9, 12	309	4	294	−
1980	3–5, 9–11	554	8	464	231
1981	3, 9	356	4	351	251
1982	1, 3	331	3	324	324
2005	2, 9	292	3	284	180
2006	2, 3, 10	351	4	325	89
2007	1–4	336	14	329	329
2008	1–4, 10–11	749	60	729	10
2009	1–4, 9	1035	75	875	92
2010	2–4, 8–10	1564	72	673	668
2011	4, 9	910	21	870	846

Herring were sampled with 25 m-long by 4 m-deep gillnets of mesh size 26 mm. The exceptions were 1979–1980 and 2005–2011 when mesh sizes ranged from 21 to 31 mm and 19–36 mm, respectively. Samples were taken throughout the year but mainly in February-April. The nets were set at the surface mainly within 1000 m from the overwintering and spawning areas of herring [42], based on local knowledge of fish distribution and/or acoustic recordings. The nets were usually set in series of three at 17:00-19:00 local time and hauled the next day (09:00-12:00). When catches were large, subsamples of up to 200 herring were analysed (see Table 1).

Herring were measured for total length (L, to the nearest 0.5 cm below, from the anterior head to the end of the caudal fin in natural position), total body weight (W, to the nearest gram), age, maturity stage and VS onboard the vessel, or were frozen immediately after capture and later measured in the laboratory. Age was determined by experienced age readers from scales until 1982 and thereafter mainly from otoliths. Eight maturity stages were distinguished based on macroscopic visual inspection of the gonads; 1–2, immature; 3–5 maturing or pre-spawning; 6, spawning/running; 7, spent; and 8, resting stage [46]. VS, the total number of vertebrae in the backbone including the urostyle, were recorded by experienced readers [46], [47]. Fulton's condition factor K [48] was calculated on the basis of autumn samples (September-November) after the feeding period as: K = 100·W/L^3^ for fish in the length range 27–31 cm, which was common for all periods.

### Data analyses

We compared basic life history parameters (age, length, age and length at first maturity, and condition factor) of herring between the three sampling periods. In cases where the life history parameters were normally distributed (length, weight and condition factor), a regular ANOVA with a Tukey HSD post-hoc test was employed to compare periods. In the case of age, the non- parametric Kruskal-Wallis test was used. The herring sampled during the three periods contained YC from five different decades (1950s, 1960s, 1970s, 1990s and 2000s) for which growth measured as length-at-age and VS could be compared independently of annual variability in sampling effort. In order to investigate length-at-age patterns at the start and end of the study period, the von Bertalanffy growth function (VBGF) [49] was fitted to length-at-age data from the YCs of the 1950 and 2000 decades using the FSA package in R. All statistics were performed using R version 3.0.0 (R Development Core Team 2013, http://www.rproject.org).

## Results

### Year class strength and longevity

**Figure 1:**
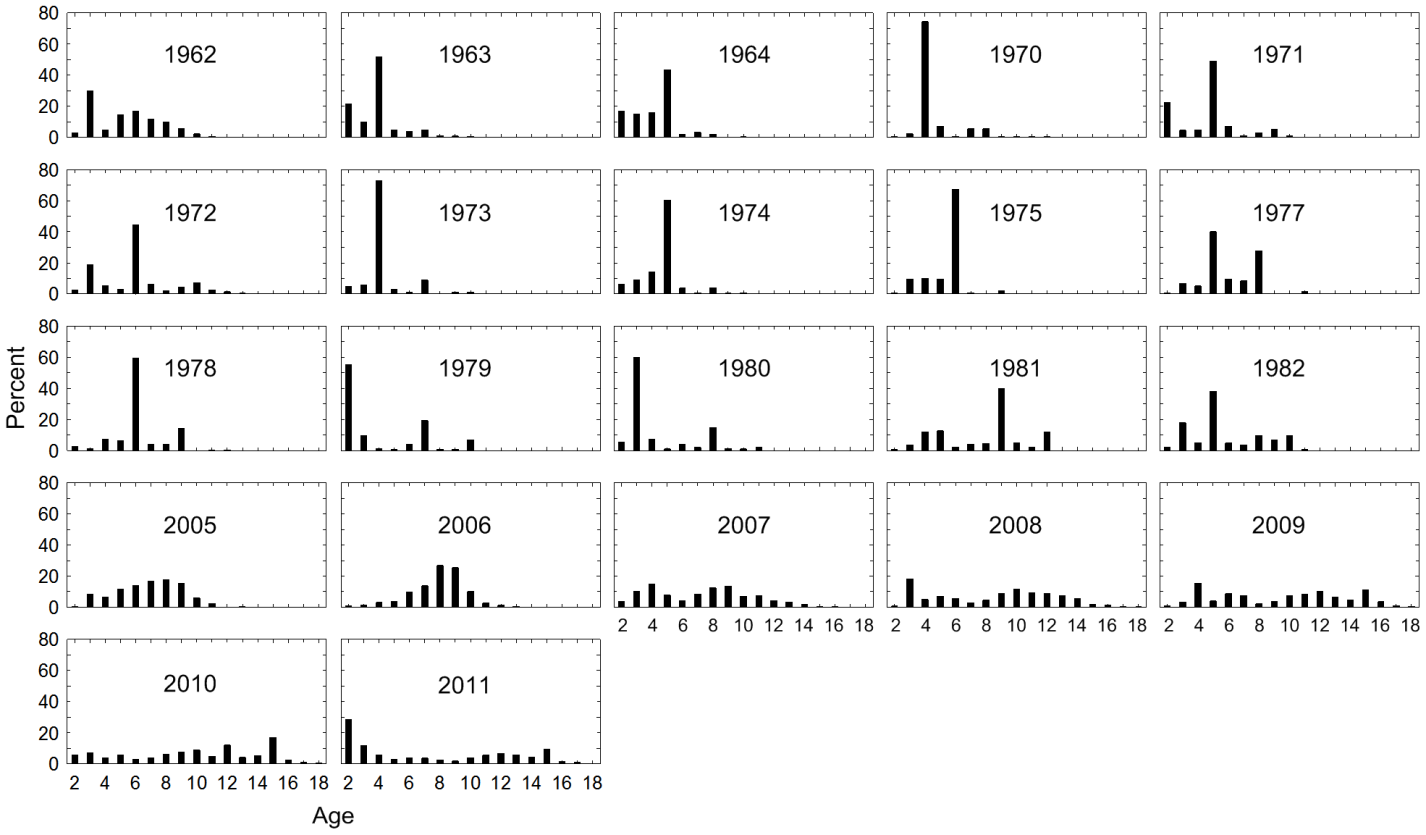
Annual age distribution. Percentages (%) by number in catches of all herring sampled 1962–2011.

In the two first sampling periods P1 and P2 (1960s and 1970–80s) the samples consisted of herring aged only up to about 10 years (Fig. 1). There were then some predominant YCs, for instance the 1969 YC that was found in the samples from age 2 (1971) to age 12 (1981). In the third sampling period P3 (from 2005) no single, predominant YC could be clearly followed over time, and from 2007 the age range became wider, and older herring (up to 20 years) were more frequent.

### Variations in life history traits over time

There were significant increases in age, length, weight and condition factor from P1 to P3 (p<0.001, Table 2). Notably, mean age and weight approximately doubled from the 1960s to the 2000s.

**Table 2:** Age, length, weight and condition factor (CF) of herring (≥2 year old) in the different sampling periods.

Parameters	P1 (1960s)	P2 (1970–80s)	P3 (2000s)
Age	4.4±1.7 (2–13)	5.4±2.3 (2–15)	8.4±4.2 (2–20)
n (Age)	1453	4789	4137
Length (cm)	25.9±2.2 (19.5–33.5)	27.2±2.7 (17.5–35.0)	31.3±2.4 (22.0–44.7)
n (Length)	1584	5059	5152
Weight (g)	134±37 (55–275)	166±53 (35–415)	256±62 (81–602)
n (Weight)	1579	4797	5119
CF	0.78±0.08 (0.51–1.22)	0.86±0.11 (0.35–1.23)	0.91±0.13 (0.28–1.22)
n (CF)	264	595	146

Values are given as mean ± SD (min-max). n indicates the number of fish sampled in the different parameters. For CF, values refer to herring in the common length range 27–31 cm for September - November.

**Figure 2:**
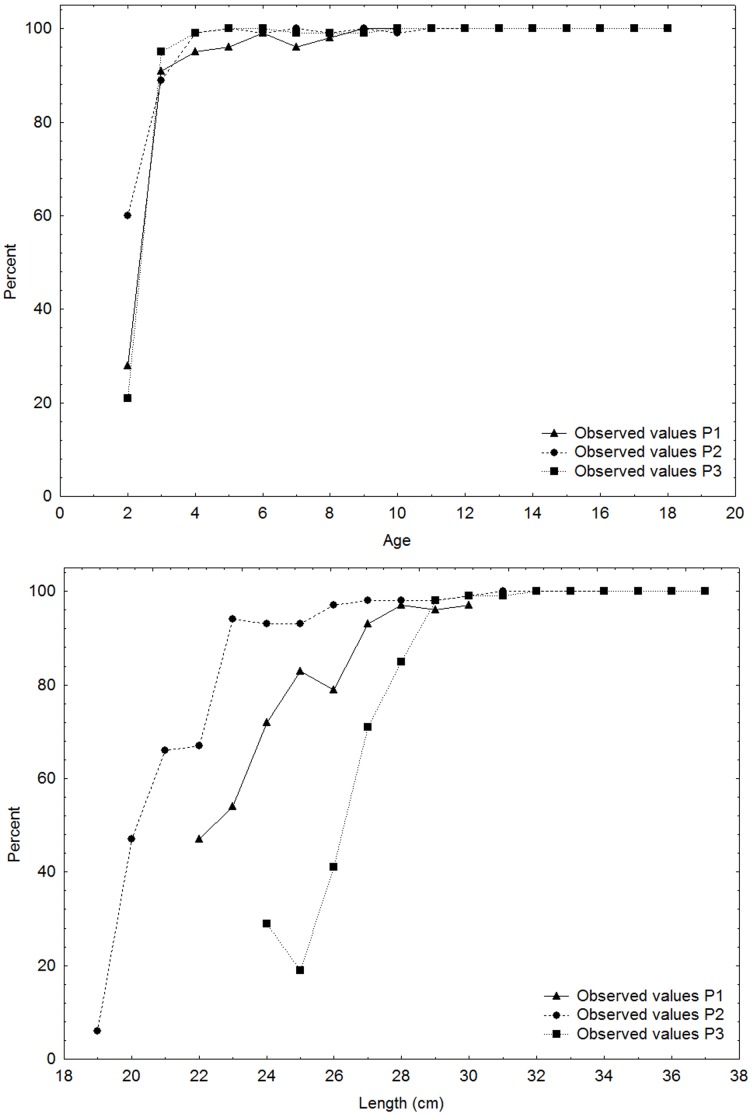
Maturity oogives. Percentages (%) of herring in stages 3–8 at (A) age and (B) length for periods P1, P2 and P3.

Age at first maturity did not change over the 50 years (Fig. 2A); in all periods the herring started to mature at age 2 and close to 100% had matured by age 3. In accordance with the increasing size over time, the length at first maturity increased from a situation where close to 100% were maturing at the length of 22–25 cm in the 1960s and 1970s to a situation where less than 20% were maturing at this length in the 2000s (Fig. 2B).

**Figure 3:**
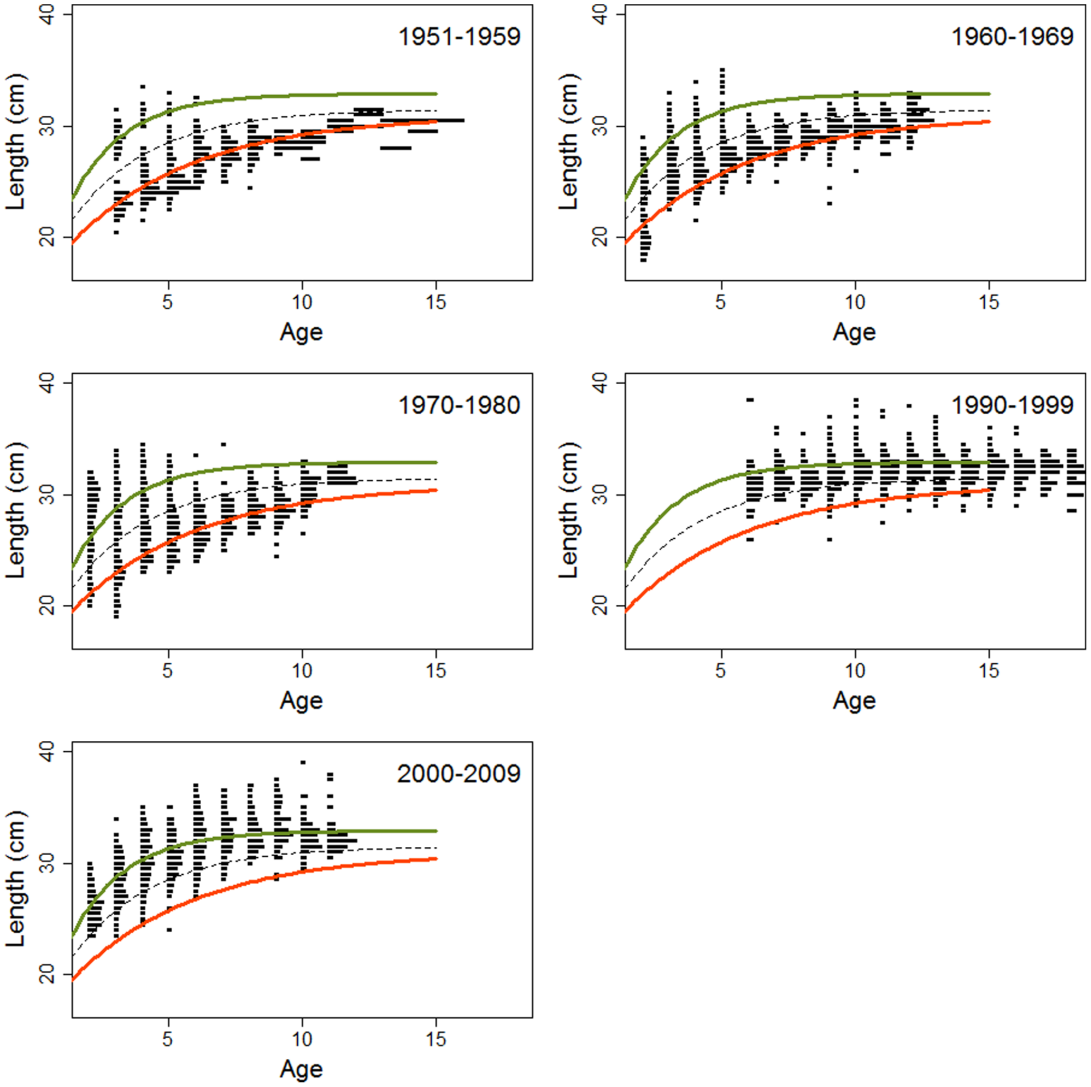
Length-at-age. Histograms averaged for herring YCs of the 1950s, 1960s, 1970s, 1990s and 2000s. A von Bertalanffy growth curve is fitted for the 1950s (red) and for the 2000s (green), and the dotted curve marks the midpoint between the two fitted curves.

**Figure 4:**
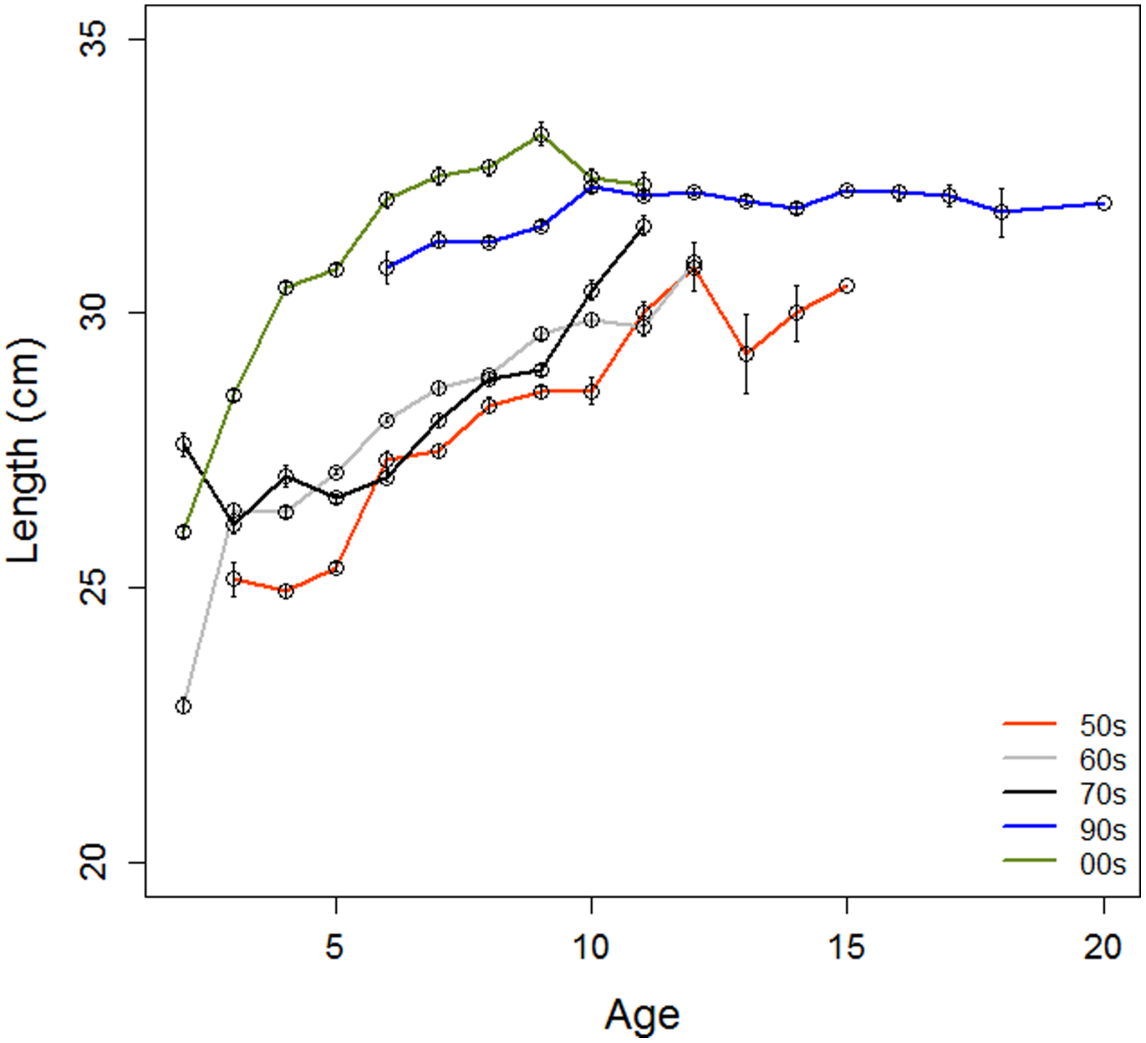
Average length-at-age. Mean ± SE of YCs of the decades 1950s to 2000s.

Figure 3 provides the length-at-age as a proxy for growth for the YCs of the 1950s, 1960s, 1970s, 1990s and 2000s. There was an increased length-at-age with decadal period (ANOVA of age groups 6–9, age groups represented in data from all decades, p<0.001, Fig. 4), with the main increase taking place between the 1970s and the 1990s. Different mesh sizes were used in the 1960s and the 2000s, but net selectivity did not explain the observed rise in length in the 2000s. When the lengths of 2–12 year-old herring (age groups present in the catches from both periods) only caught with 26 mm mesh size were compared between the two sampling periods, there was still a marked difference in length (1960s: mean 25.9 cm±2.2, 2000s: 30.9 cm±2.6 (p<0.001).

### Occurrence of different herring components

A visual inspection of the length-at-age plots in Figure 3 strongly suggest that two growth modes were present in young herring (<6 years) in the decades 1950–1970. In contrast, at ages ≥6 years the fast growing mode was hardly observed. In order to compare length-at-age at the start and end of the study period, two growth curves were fitted with the VBGF (see Materials and Methods). The first was based on mean length-at-age for each age group in the 1950s YCs (red superimposed curve in Fig. 3), but excluding the rapid-growing mode of the 2–5 year-olds. The second was based on mean length-at-age for each age group in the 2000s YCs (green curve). The two growth curves clearly differed. The green curve of the 2000s had a steeper increase (k = 0.49 versus 0.22), indicating a faster growth rate of young fish, and reached the asymptote at a higher level (L_∞2000YCs_ = 329 versus L_∞1950YCs_ = 310) than the red curve of the 1950s, indicating increased maximum length. The red slow-growing curve fitted to the 1950s YCs corresponds to a slow growth pattern expected of resident herring, while the green curve fitted to the 2000s YCs is more similar to the NSS growth pattern. Notably, the young herring in the 1950s–1970s with rapid growth displayed growth patterns that fitted better with growth of the 2000s YCs (Fig. 3).

The connectivity between the two components changed with fish age. The proportion of fast growers decreased with age for the young herring from 0.59 for 2-year-olds to 0.12 for 5-year-olds (Table 3). Furthermore, the decrease was steeper during P1 than P2, indicating that the rapid-growing fish remained for longer in the system during P2.

**Table 3:** Proportion of rapid growers for herring in age groups 2–5, in the sampling periods P1 (1960s) and P2 (1970–1980s).

Age	Prop fast total	Prop fast P1	N	Prop fast P2	N
2	0.59	0.74	223	0.52	476
3	0.52	0.56	245	0.50	634
4	0.26	0.19	342	0.30	582
5	0.12	0.04	391	0.14	1199

The two growth modes were not easily distinguishable during the sampling period P3.

**Figure 5:**
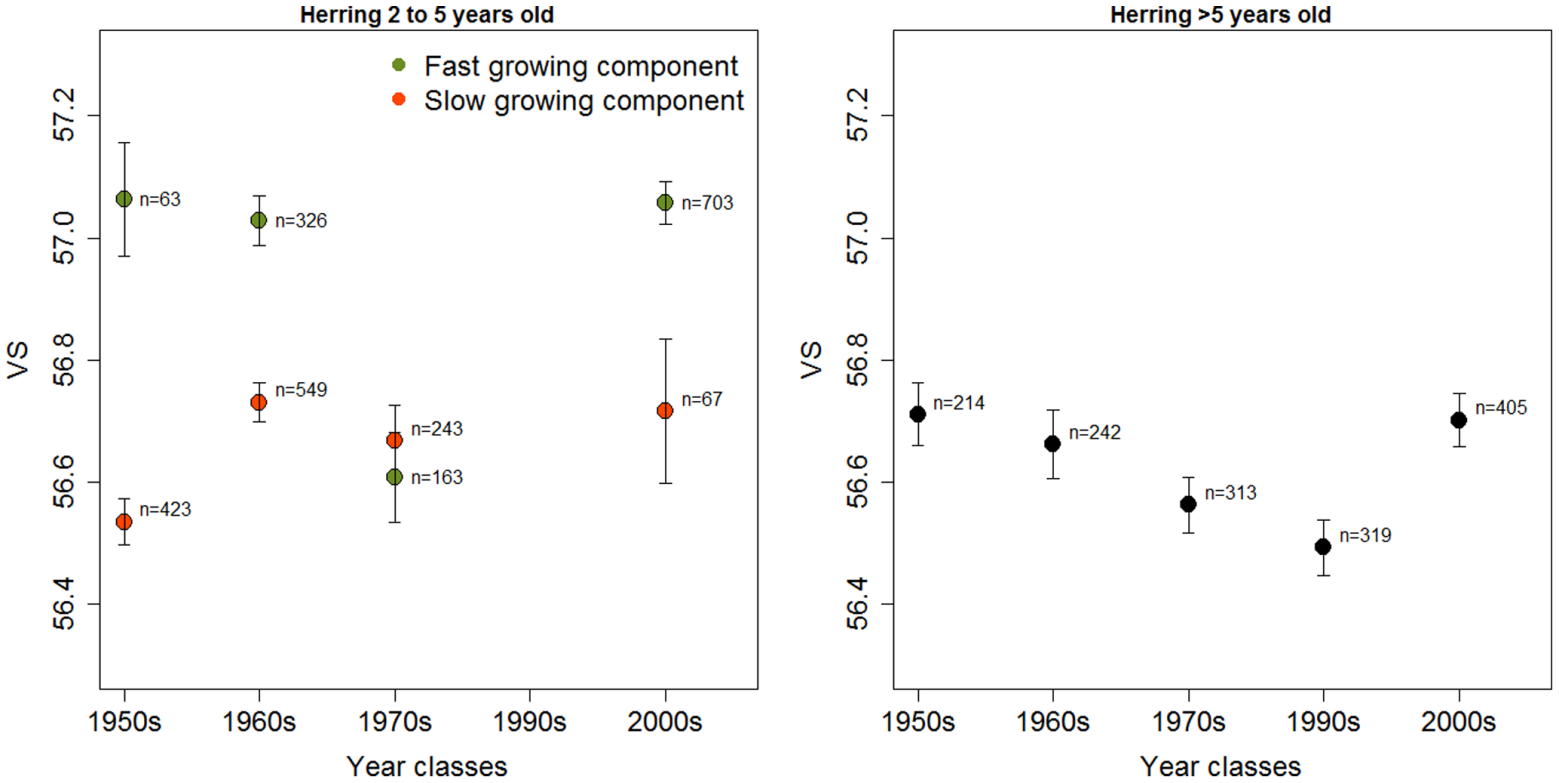
VS of young (2–5 years old) and older (6–11 years old) herring. Mean VS ± SE of YCs of the decades 1950s to 2000s. Individuals with length-at-age closest to the 1950s slow-growing curve shown in red and the ones closest to the 2000s fast-growing curve shown in green. Sample size is given for the different components.

In order to determine whether there was any difference in VS between rapid- and slow-growing fish indicating different areas of origin, VS was compared between fish with length-at-age closest to the VBGF fitted to the 1950s (slow-growers, red curve in Fig. 3) and the 2000s (fast-growers, green curve in Fig. 3). Comparisons were made for young (2–5 years old) herring for all decades for which data were available (not the 1990s). Figure 5 shows that fast-growing young herring in the 1950 and 1960 YCs had higher VS (t-test, p<0.001) than slow-growing fish of the same YCs. This indicates that these two groups of young herring originated from different areas and thus represent different populations, presumably NSS and LP herring. On the other hand, for young herring of the 1970 YCs, the VS of the rapid and slow growing components overlapped (t-test, p = 0.93), indicating that they originated from the same area. The 2000 YCs showed the same tendency as the 1950 and 1960 YCs, with higher VS for fast-growers than slow-growers (p = 0.08), but the presence of two growth modes was not clear among the young herring for the 2000 YCs so this method of comparing different growth modes is less adequate. For the older fish (6–11 years old) VS was in general similar to VS of the slow-growing young herring (about 56.6, Fig. 5) indicating a dominance of resident herring of local origin.

### Overlap in spawning time between different components of herring

**Figure 6:**
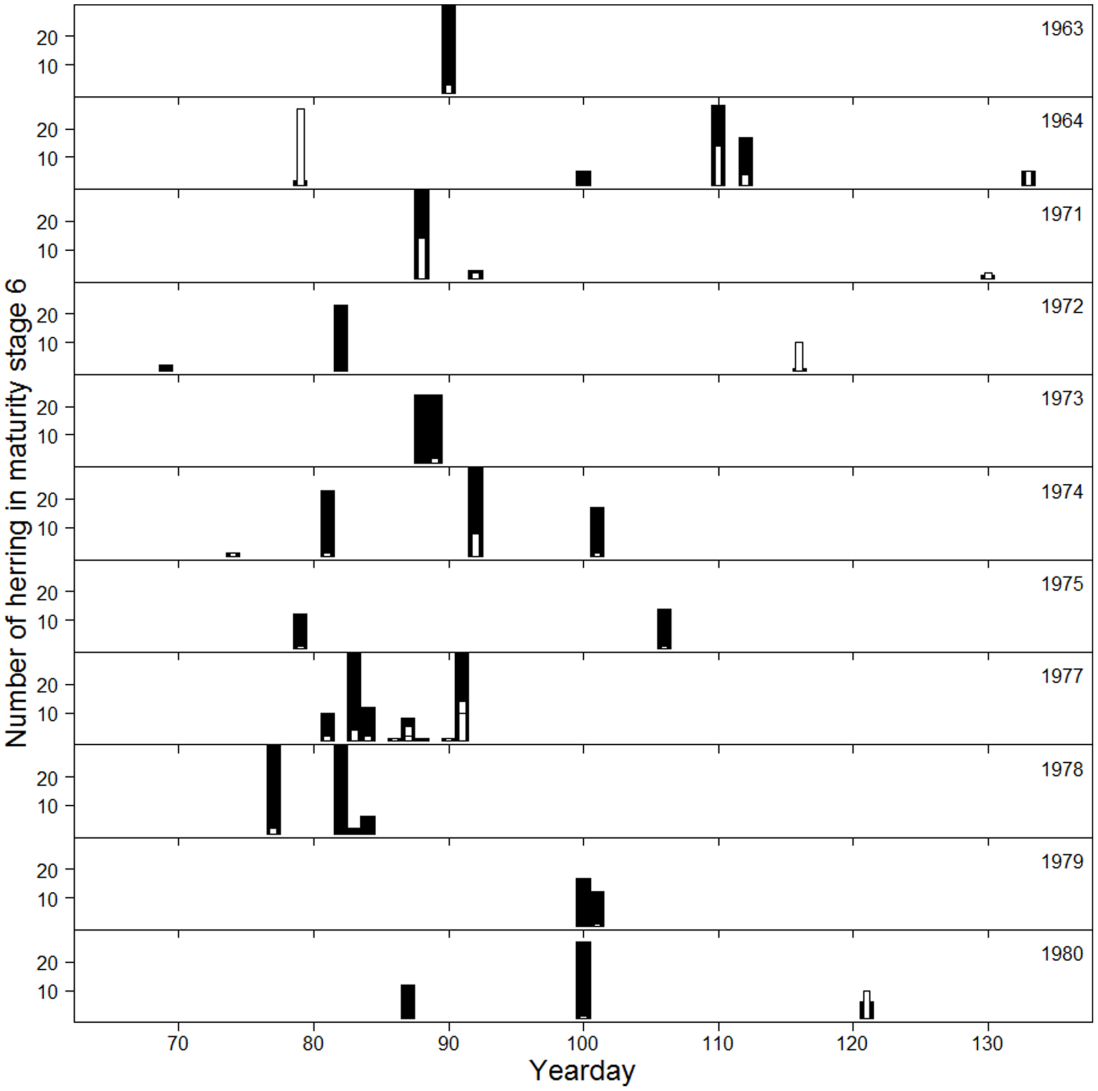
Timing of spawning in fast and slow growers. Number of fast (white) and slow growers (black) in stage 6 (spawning stage) as a function of yearday, all stations in P1 and P2 are included. For the sake of clarity, there is a cut-off on the y-axis at 30 individuals.

A prerequisite for the existence of a metapopulation is that there is an overlap in timing of spawning between populations. To investigate the overlap between the two components identified, all fish in maturity stage 6 (spawning) were extracted from the data from P1 and P2 (Fig. 6, P3 excluded due to less certain discrimination of the components). Most samples contained herring from both components. Since the proportion of the rapid-growing component was usually lower, and most samples therefore only contained a limited number of this component, an additional comparison was made, including only stations at which at least 10 fish from both components were present in stage 6. In the four cases that fulfilled these criteria, the overlap was close to complete. The findings strongly suggest that spawning among the components was synchronized.

**Figure 7:**
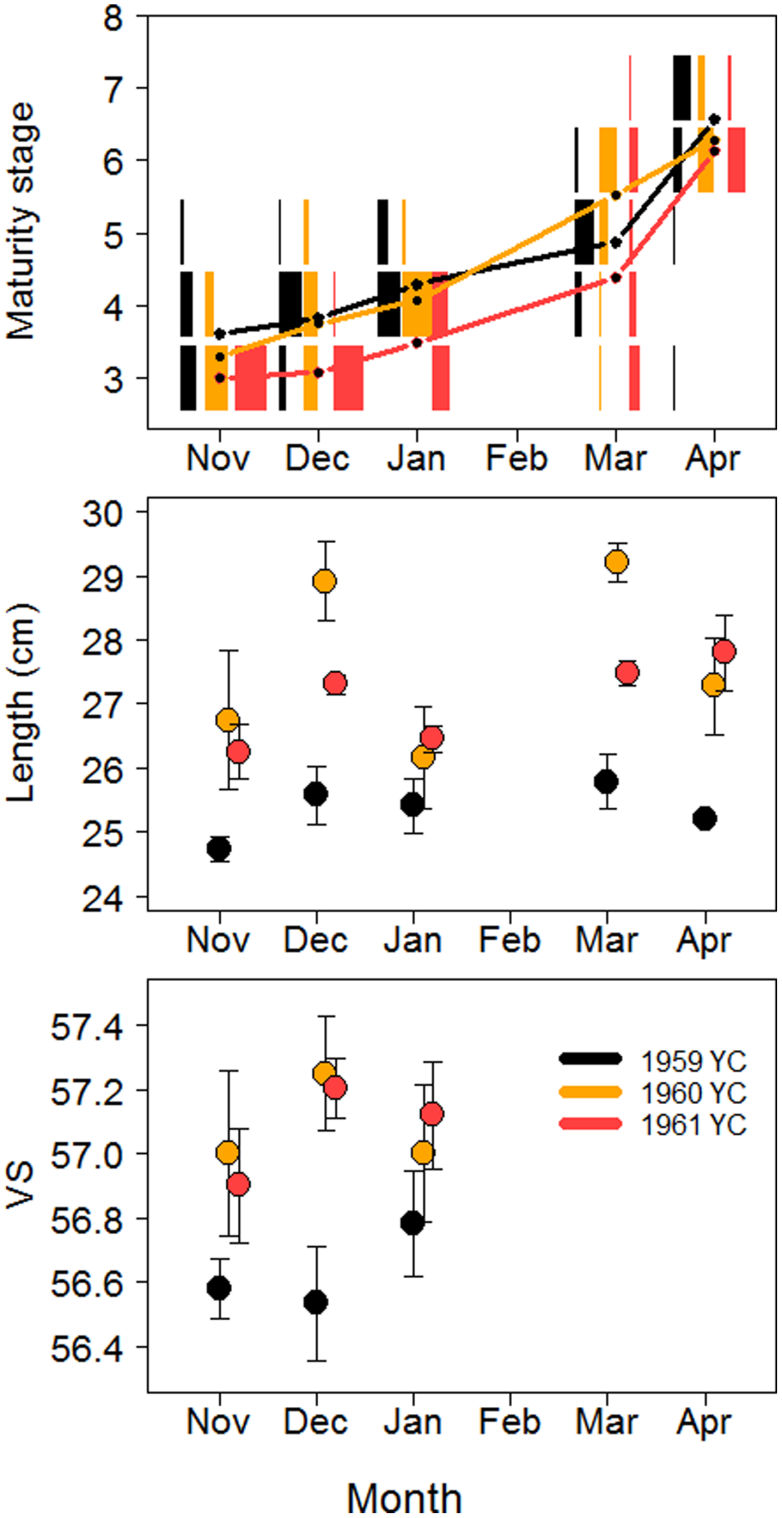
A-C. Monthly mean values of maturity stage, length and VS of herring. Values are given for the period November 1963 through April 1964. Relative monthly proportions of maturity stages are given for each of the 1959–61 YCs.

A small part of the data set could be used to resolve the temporal development of maturation on a finer scale. Between November 1963 and April 1964, the herring were sampled throughout the maturation period. Figure 7A shows that YCs 1959, 1960 and 1961 (3–5 years old spawners where two components should be present) had similar maturation patterns, with a large majority of the fish from all YCs in stage 6 (‘spawning’) or 7 (‘spent’) in April. There was no falling trend in length during the maturation period (Fig. 7B), as would have been expected if the fast-growing NSS herring left before spawning. The finding that the 1959 YC, despite being the oldest, generally had a smaller length and lower VS than the other YCs (Figure 7B, C) supports the idea that fast-growing NSS herring disappear from the area at a certain age.

## Discussion

This is the first documentation of a long-term dynamic relationship between populations in a pelagic fish species during reproduction. We have found that a component of Atlantic herring inferred to belong to the resident LP herring population co-occurred in its coastal fjord home range with a herring component assumed to belong to the much larger population of Norwegian spring spawning (NSS) herring. The components remained together during spawning, with synchronised maturation states. Over a period of 50 years the resident herring underwent substantial changes in several life-history traits, including length-at-age, length at first maturity and longevity. The observed connectivity and mixed spawning between the components may be the drivers of the changes in life-history traits in the initially slow growing and relatively short-lived resident herring.

### Herring components in Lindåspollene

VS are primarily determined by the environmental conditions during the embryonic stages [18], [50], [51] and thus have the potential to distinguish components of the herring that display different distribution and migration patterns. A central assumption in the interpretation of the data was that herring of coastal origin have lower VS than oceanic herring. A low VS and small size are characteristics of resident herring populations on the Norwegian coast [14], [19], and the component with slow growth and low VS in Lindåspollene in the 1960s and the 1970–1980s was thus classified as belonging to the resident Lindås (LP) herring population. Oceanic NSS herring are characterized by rapid growth and high VS ([10], [11], [17], [21], and young fish with these characteristics in the 1960s (P1) and 2000s (P3) were thus classified as belonging to the oceanic NSS population.

Classifying the herring components with different characteristics than the ones described above, is not a straightforward process. In the 1970–1980s (P2) the rapid-growth component also had low VS. The population of NSS herring had by then nearly collapsed, and the remaining NSS herring stayed close to the coast throughout the year [23], [52], [53]. Spawning occurred more inshore than on the traditional spawning grounds on the large banks, with the vast majority of the progeny hatching in coastal areas [25]. The low VS suggests that NSS herring then utilised similar spawning areas as the resident populations [54]. We therefore assume that this component is NSS herring of coastal origin. In the 2000s, when the population of NSS herring had recovered, VS again increased in rapidly growing young fish (Figure 5). The decrease and subsequent increase in VS in young fish are in synchrony with the decline and rise of the oceanic NSS herring. In older fish, VS were generally low, supporting the notion that relatively few old oceanic NSS herring were present. Interestingly, the slow-growing component of the young fish with low VS had now been strongly reduced and a fast-growing component with low VS dominated. This component is interpreted as resident fast-growing herring. The greatest increase in length-at-age took place from the 1970s to 1990s YCs. Most herring in Lindåspollene in the 2000s (P3) were thus presumably rapid-growing oceanic NSS herring and resident herring (Figures 3 and 5). Any remaining old NSS herring of coastal origin could not be distinguished from fast-growing resident herring, but genetic analyses have demonstrated that herring in Lindåspollene in the 2000s differ from NSS herring (Christophe Pampoulie, Marine Research Institute Iceland, unpublished observations). In the future, genetic analyses can be used to investigate the rate and extent of genetic change due to interbreeding.

The proportion of the component classified as NSS herring in Lindåspollene diminished markedly with age, with the proportion of five-year-old NSS herring only a fifth of the proportion of two-year-olds (Table 3). The NSS herring thus seem to enter the system as young or at the larval stage and spawn one or a few times with the resident herring. But why do they then leave the area? Size may be a threshold trait that determines migratory tactics [55], and herring above a certain size may migrate to more productive habitats, as has been observed in salmonids [56], [57]. In the 2000s, resident herring of similar size as NSS herring remained in the system, but a genetic influence (see [58]) and different previous experience could result in different migration thresholds in LP and NSS herring. The decrease in proportion of NSS herring with age was strongest in P1, suggesting that NSS herring stayed longer in the system during P2 than P1. The chance of interbreeding between the resident and NSS herring could thus be higher during P2.

### Interbreeding between NSS and LP herring

So do fish classified as NSS herring actually spawn with fish classified as LP herring? In the 1963–1964 data, no decrease in size during the course of the maturation period was observed, and the larger NSS herring thus seem to stay in Lindåspollene throughout spawning. Most importantly, young NSS and LP herring at maturation stage 6 (spawning), were caught together in the same gillnet settings. This stage of maturity typically lasts only for a day or so (Olav Kjesbu, Institute of Marine Research, pers. comm.). In addition, echosounder recordings and data from acoustic tags during spawning in Lindåspollene provide no evidence that herring split into groups according to origin [59], [60]. Altogether, the evidence strongly indicates that LP herring and NSS herring interbreed in Lindåspollene.

### Changes in life-history traits in LP herring

We have clearly demonstrated that several life-history traits in LP herring changed over time, with the mean age and weight of this component rising by almost 100% from the 1960s (P1) to the 2000s (P3), but it is not straightforward to explain these changes. There is no information available to suggest that changes have taken place in relevant environmental conditions in Lindåspollene. The marked changes in life-history traits could be a consequence of phenotypic plasticity, genetic responses or a combination of the two [61]. Herring is a flexible species with a high level of adaptability as a basic trait [62]. The genetic structure of life-history traits and high genetic diversity may provide a flexible species with large phenotypic plasticity and/or a large potential for rapid evolutionary changes [62], [63].

The heritability of life-history traits is quite large [64], and interbreeding with NSS herring might well have changed genetically determined life-history traits in LP herring from slow growth and short life-span to faster growth and a longer life. Fish have limited energetic resources and there are trade-offs between somatic growth, survival and reproduction [65]–[67]. Rapid growth is crucial for the highly migrating NSS herring as the energy costs of swimming decrease as size increases [29]. For the more stationary LP herring large size is probably less critical. The trade-off between growth and reproduction is generally strong in herring [68], [69]. LP herring should thus be expected to have invested more in reproduction when they were small early in the study period, and unpublished observations from the 1970s in fact indicate a high reproductive effort (RE). We would therefore expect that the RE of LP herring in the 2000s was similar to NSS herring, as both LP and NSS herring then had fast growth. However, RE was found to be considerably higher in LP than NSS herring also in the 2000s [20]. What is genetically determined is not a set value but a reaction norm [70], and by migrating to more productive outside waters (see below), LP herring could obtain more resources with low energy costs and thereby combine rapid growth and longevity with high RE.

McQuinn [6] argued that the structure of herring populations is of a behavioural rather than genetic nature. Although there are few data on movements of LP and NSS herring in and out of Lindåspollene, it is reasonable to assume that a change in feeding migration patterns was involved in the increased growth of LP herring. Migration patterns in herring are presumably maintained by local traditions [29]–[32], and pre-spawning LP herring have aggregated in a particular area in Lindåspollene for several decades [12], [59], [71]. By social transmission of migration patterns (see also [72]), NSS herring may have triggered a change in LP herring to migrating to more productive outside waters, leading in time to increased growth. As there is a genetic basis for migratory behaviour [73], [74], interbreeding with migratory NSS herring may also have increased the tendency of LP herring to migrate. All in all, it is reasonable to assume that interactions between genetic factors that influence both growth potential and migratory tendency, and changes in migration traditions, are involved in the change of life- traits in LP herring.

## Conclusions and Perspectives

We have presented new evidence that Atlantic herring form a metapopulation, and for the first time have found evidence of strong temporal dynamics between two herring populations. However, although the data material is unique and our explanations are plausible, we recognise that some questions remain to be answered. The application of VS is useful in the lack of more sophisticated identification methods such as genetic analyses, but due to the large individual variation in VS also within a single herring component, relatively large samples would be needed to obtain a robust basis for comparison. Nor is it clear whether most NSS herring initially enter Lindåspollene at the larval stage or later, and there are few data on movements of LP and NSS herring in and out of Lindåspollene. We have generally strong evidence that NSS herring leave Lindåspollene after a certain age and size, but this was more difficult to study in the 2000s with our methodology as LP and NSS herring were similar in size. This demonstrates the limitations of using VS and growth patterns to distinguish different components.

When NSS herring underwent a distributional shift after the stock collapse, the connectivity with resident herring could have led to changes in several life-history traits of the indigenous herring by interbreeding and/or a culturally mediated migratory shift. A metapopulation of herring could consist of populations that differ genetically but that also possess cultural diversity, such as different migration patterns, as an additional isolating mechanism. But migration routes can change. Historical records indicate that NSS herring have undergone large fluctuations in abundance during the past 500 years ([75] and references therein) causing changes in migration patterns [23]. Our finding that LP herring became more similar to NSS herring over the study period may suggest a cyclical pattern with genetic differentiation of the resident population during periods of weak connectivity resulting in slower growth and shorter life span alternating with periods of strong connectivity that reduce these differences.

Changes in migration patterns may be associated with a fishery-induced collapse [26]. This study suggests that anthropogenic influences such as a fishery should not be considered in isolation for a single herring population. Exploitation of neighbouring populations may alter the composition of the metapopulation in ways that could challenge the integrity of the resident population, and high rates of connectivity between populations could decrease the productivity and stability of a population [76]. Further studies of dynamic interchange between different populations of marine species and the relative importance of genetic and cultural factors behind the population structure are warranted, both from a basic ecological and evolutionary perspective and as a basis for sound management.
